# Rare Genetic Variants in Human *APC* Are Implicated in Mesiodens and Isolated Supernumerary Teeth

**DOI:** 10.3390/ijms24054255

**Published:** 2023-02-21

**Authors:** Chomchanok Panyarat, Siriruk Nakornchai, Kanoknart Chintakanon, Niramol Leelaadisorn, Worrachet Intachai, Bjorn Olsen, Sissades Tongsima, Ploy Adisornkanj, Chumpol Ngamphiw, Timothy C. Cox, Piranit Kantaputra

**Affiliations:** 1 Center of Excellence in Medical Genetics Research, Faculty of Dentistry, Chiang Mai University, Chiang Mai 50200, Thailand; 2 Division of Pediatric Dentistry, Department of Orthodontics and Pediatric Dentistry, Faculty of Dentistry, Chiang Mai University, Chiang Mai 50200, Thailand; 3 Department of Pediatric Dentistry, Faculty of Dentistry, Mahidol University, Bangkok 10400, Thailand; 4 Division of Orthodontics, Department of Orthodontics and Pediatric Dentistry, Faculty of Dentistry, Chiang Mai University, Chiang Mai 50200, Thailand; 5 Theera-Niramol Dental Clinic, Roi-et 45000, Thailand; 6 Department of Developmental Biology, Harvard School of Dental Medicine, Boston, MA 02115, USA; 7 National Biobank of Thailand, National Science and Technology Development Agency (NSTDA), Thailand Science Park, Pathum Thani 12120, Thailand; 8 Dental Department, Sawang Daen Din Crown Prince Hospital, Sakon Nakhon 47110, Thailand; 9 Departments of Oral & Craniofacial Sciences, School of Dentistry, and Pediatrics, School of Medicine, University of Missouri-Kansas City, Kansas City, MO 64108, USA

**Keywords:** Extra teeth, mesiodentes, Gardner syndrome, supernumerary tooth, WNT signaling, taurodontism, root maldevelopment

## Abstract

The activation of Wnt/β-catenin signalling is a prerequisite for odontogenesis. APC, a member of the AXIN-CK1-GSK3β-APC β-catenin destruction complex, functions to modulate Wnt/β-catenin signalling to establish regular teeth number and positions. APC loss-of-function mutations are associated with the over-activation of WNT/β-catenin signalling and subsequent familial adenomatous polyposis (FAP; MIM 175100) with or without multiple supernumerary teeth. The ablation of Apc function in mice also results in the constitutive activation of β-catenin in embryonic mouse epithelium and causes supernumerary tooth formation. The objective of this study was to investigate if genetic variants in the *APC* gene were associated with supernumerary tooth phenotypes. We clinically, radiographically, and molecularly investigated 120 Thai patients with mesiodentes or isolated supernumerary teeth. Whole exome and Sanger sequencing identified three extremely rare heterozygous variants (c.3374T>C, p.Val1125Ala; c.6127A>G, p.Ile2043Val; and c.8383G>A, p.Ala2795Thr) in *APC* in four patients with mesiodentes or a supernumerary premolar. An additional patient with mesiodens was compound as heterozygous for two *APC* variants (c.2740T>G, p.Cys914Gly, and c.5722A>T, p.Asn1908Tyr). Rare variants in *APC* in our patients are likely to contribute to isolated supernumerary dental phenotypes including isolated mesiodens and an isolated supernumerary tooth.

## 1. Introduction

Supernumerary teeth refer to extra teeth that exceed the usual number in deciduous or permanent dentitions [1,2,3], though they are rare in deciduous dentition [2,4]. Supernumerary teeth may occur as a single tooth or multiple teeth in any region of the maxilla, premaxilla, and mandible in the same person [1,5]. The prevalence of supernumerary teeth has been reported to be 0.04–3.8% [1,3,5,6,7]. However, supernumerary teeth located in the premaxilla region (referred to as mesiodens) are the most common form, with an estimated prevalence ranging from 0.15% to 1.9%, depending on the method of selecting the samples and the studied population [8,9,10,11]. Interestingly, supernumerary teeth are more prevalent in Asian populations [5,12,13]. However, as supernumerary teeth can remain unerupted, the true incidence is likely to be under-reported as radiography is not used in all dental assessments [6].

Wnt/β-catenin signalling is involved in almost every aspect of embryonic development and also regulates homeostatic self-renewal in tissue regeneration and repair. Wnt/β-catenin signalling plays a particularly critical role in the development of ectodermally derived organs, including skin, hair, sweat glands, nails, and teeth [14,15]. Indeed, defects in the components of the Wnt signaling pathway (eg. WNT10A and WNT10B) that lead to reduced WNT signalling strength are the most common causes of tooth agenesis [14,15,16,17,18].

Recently, mutations in *LRP5, LRP6*, *WLS, DKK1*, and *LRP4* have been reported to be predisposing factors for mesiodens in humans [19,20,21,22,23]. Supernumerary incisors have also been reported in various mouse knockout lines, including mutants of Wnt pathway receptors (*Lrp4*) and inhibitors (*Wise*) [24,25]. Supernumerary teeth have also been reported to be associated with various malformation syndromes, including *APC*-associated familial adenomatous polyposis (FAP) [2,26,27], *RUNX2*-associated cleidocranial dysplasia [27,28,29], and *TRPS1*-associated tricho-rhino-phalangeal syndrome [27,30,31,32,33].

The *APC* gene (MIM 611731) encodes a multidomain tumour-suppressor protein that, as described above, has an important role in assembling the AXIN-CK1-GSK3β-APC destruction complex that modulates the level of WNT/β-catenin signalling [34,35]. APC loss-of-function mutations are associated with over-activation of WNT/β-catenin signalling and subsequent FAP (MIM 175100) or Gardner syndrome [26], characterized by adenomatous polyposis of the colon, multiple supernumerary teeth, craniofacial osteomas, epidermal cysts, congenital hypertrophy of the retinal pigmented epithelium, and desmoid tumours [26,36]. The ablation of the Apc function in mice also results in the constitutive activation of β-catenin in embryonic mouse epithelium and causes supernumerary tooth formation [37,38,39]. Intriguingly, only 11–27% of patients with familial adenomatous polyposis have supernumerary teeth with no genotype-phenotype correlation [2]. The objective of this study was to investigate if genetic variants in the *APC* gene were associated with supernumerary tooth phenotypes.

Here, we report five rare variants in *APC* in six patients with isolated mesiodentes and a patient with an isolated supernumerary premolar. To the best of our knowledge, this is the first report demonstrating that variants in *APC* are implicated in isolated supernumerary tooth formation.

## 2. Results

**Figure 1:**
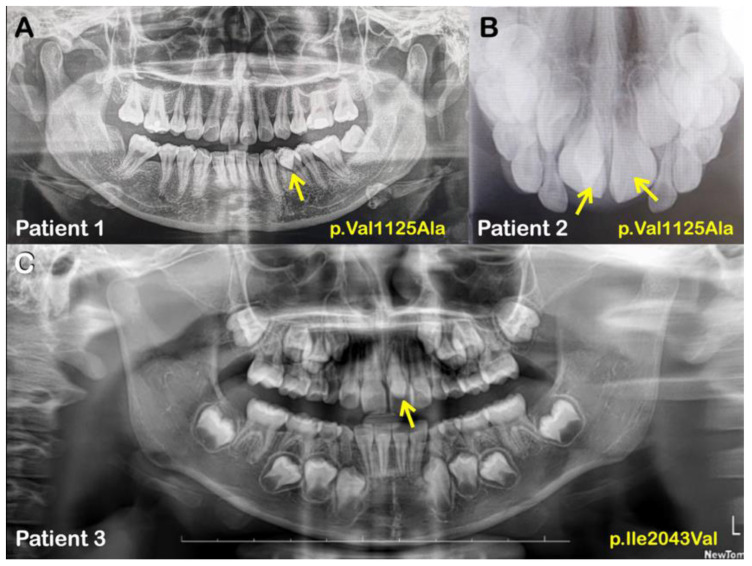
(**A**) Patient 1. Panoramic radiograph showing a supernumerary left mandibular premolar (arrow). (**B**) Patient 2. Occlusal radiograph showing double mesiodentes (arrows). (**C**) Patient 3. Panoramic radiograph showing a mesiodens (arrow).

**Figure 2:**
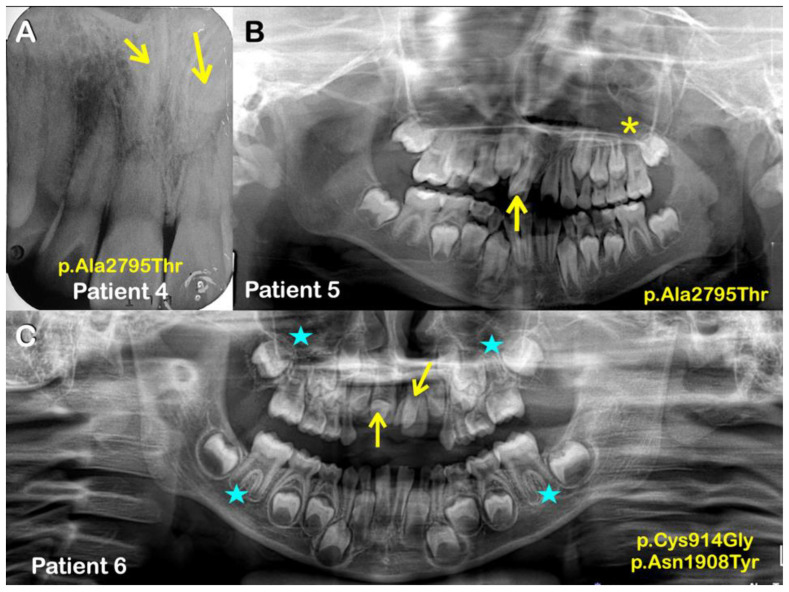
(**A**) Patient 4. Periapical radiograph showing double mesiodentes (inverted) (arrows). (**B**) Patient 5. Panoramic radiograph showing a mesiodens (arrow) and taurodontism (asterisk). (**C**) Patient 6. Panoramic radiograph showing double mesiodentes (arrows) and taurodontism (asterisks).

**Figure 3:**
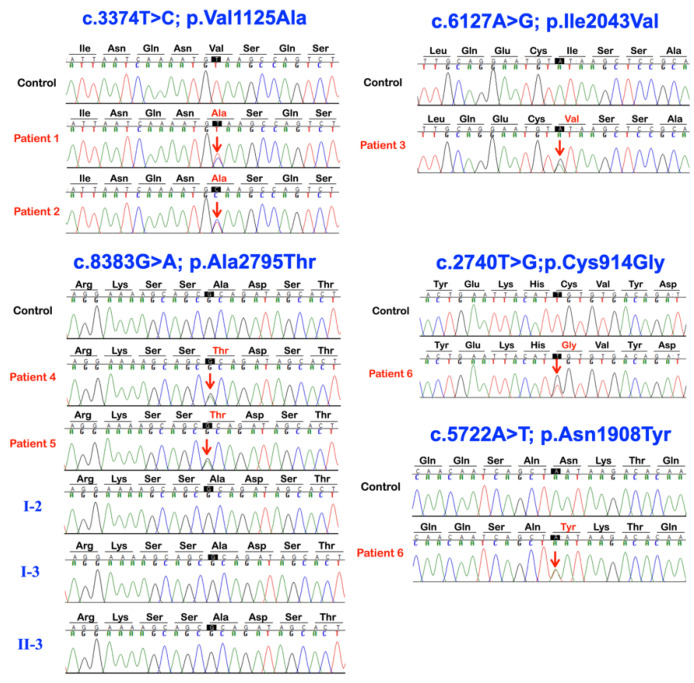
Sequence chromatograms of *APC* variants in patients 1–6, controls, and their unaffected family members.

We identified five APC missense variants in five unrelated patients (plus one affected family member in family 4) presenting with either isolated mesiodentes (five cases) or an isolated supernumerary premolar (one case) (Table 1; Figure 1 and Figure 2). All the variants were validated using Sanger sequencing and, in the case of family 4, the variant was present in both the affected siblings but not in the unaffected sibling and unaffected mother (Figure 3).

**Figure 4:**
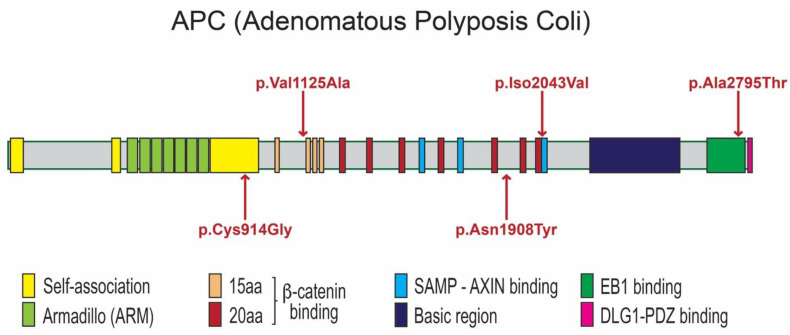
Protein domains of APC proteins. APC is comprised of regions required for oligomerization, Armadillo repeats, β-catenin-binding domain, AXIN-binding domain, Basic domain, EB1-binding domain, and HDLG-binding domain. Locations of mutations are indicated by red arrows.

A heterozygous missense variant (c.3374T>C; p.Val1125Ala; rs377278397; MAF: 0.0005993) was found in two unrelated patients—one (patient 1) with double mesiodentes and the other (patient 2) with the extra premolar. The CADD score of the p.Val1125Ala variant is 21.7 and it is predicted to be disease causing according to MutationTaster. The amino acid residue Val1125 is located immediately before the second of the four 15-amino acid repeat motifs (Figure 4), which are required for binding to the C-terminal Binding Protein (CTBP; MIM 602618) [40].

**Figure 5:**
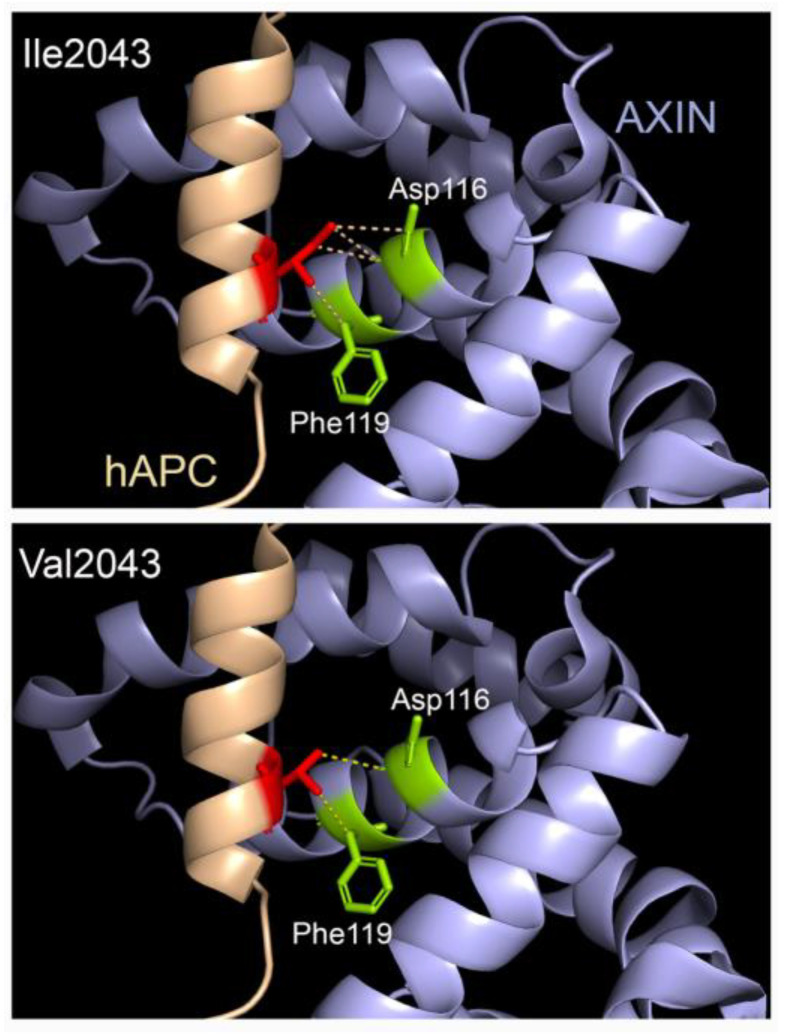
The Ile2043Val missense variant in the SAMP3 domain of APC may impact the strength of interaction with AXIN. Human APC SAMP3 domain is shown in beige while the RGS domain of AXIN is coloured mauve. Isoleucine 2043 in wildtype human APC has four contacts with the AXIN RGS domain: three with Asp116 and one with Phe119. The valine substitution in APC is predicted to reduce the contacts with Asp116 to just one.

An additional rare variant, c.6127A>G; p.Ile2043Val (rs876660233; MAF: 0.000007085), was identified in the third individual (patient 3). The CADD score of p.Ile2043Val is 24.9 and it is predicted to be disease causing, probably damaging, and damaging by MutationTaster, PolyPhen-2, and SIFT, respectively. The p.Ile2043Val variant is located within the SAMP3 motif, which is critically required for interaction with the RGS domain of AXIN (Figure 4 and Figure 5). Residue Ile2043 is one of only a few invariant residues in the SAMP domains and is one of nine residues that have a direct contact with AXIN, specifically with Asp116 and Phe119 within the a4 helix of the AXIN RGS domain [41].

The third variant, c.8383G>A; p.Ala2795Thr (rs369264968; MAF: 0.00004400), was found in two siblings of family 4 (patients 4 and 5)—each with double mesiodentes—but not in the third sibling that had normal dentition (Figure 1 and Figure 2; Table 1). The CADD score of the p.Ala2795Thr variant is 22.2 and it is predicted to be disease causing and probably damaging by MutationTaster and PolyPhen-2, respectively. The p.Ala2795Thr variant is located toward the C-terminus of the APC protein in a region required for interaction with the End-Binding 1 protein (EB1; MIM 603108), which targets APC to microtubule plus-ends [42].

The sixth patient (patient 6), who had double mesiodentes, was found to be compound heterozygous for two variants: c.2740T>G; p.Cys914Gly and c.5722A>T; p.Asn1908Tyr. Neither variant was reported in gnomAD or LOVD, although the former is reported in NCBI with reference number: rs1554084426. The p.Cys914Gly variant is located between the armadillo repeat domain and the 15 amino acid actin and β-catenin binding repeats (Figure 4). This inter-repeat region is one of three segments of the APC N-terminus that has self-association properties and is thus thought to be involved in the oligomerization of APC. The CADD score of the p.Cys914Gly variant is 21.5 and it is predicted to be disease causing by MutationTaster. The p.Asn1908Tyr variant (CADD score: 17.19) is located within the overlapping region required for both b-catenin and AXIN binding (Figure 4). Specifically, the variant lies between the 20 amino acid repeat 5 and 6 that contribute to b-catenin binding and are located between the second and third SAMP domains (AXIN binding). It is predicted to be damaging by SIFT. 

## 3. Discussion

The WNT/β-catenin, BMP, and SHH signalling pathways play important interrelated roles in the process of tooth development, with mutations in components of the Wnt/β-catenin pathway arguably being the most common cause of dental anomalies [15,19,20,21,43]. The downregulation of WNT/β-catenin is implicated in both tooth agenesis and microdontia [19,20,21,43,44], while the over-activation of WNT/β-catenin and SHH signalling is implicated in supernumerary tooth formation [7,27,31]. 

The activation of WNT/β-catenin signalling is initiated when a WNT ligand binds to a Frizzled (FZD) receptor and a coreceptor, LDL receptor-related protein 5 or 6 (LRP5/6), forming a WNT-FZD-LRP5/6 complex [16,45,46]. The WNT-FZD-LRP5/6 complex subsequently recruits a ‘destruction complex’ consisting of AXIN, Casein kinase 1 (CK1), Glycogen synthase kinase 3β (GSK3β), and Adenomatous polyposis coli (APC). This recruited AXIN-CK1-GSK3β-APC complex interacts with the intracellular domains of FZD-LRP5/6 at the plasma membrane through an adaptor protein Disheveled (DVL) and subsequently becomes inactivated. Once the destruction complex is inactivated, the phosphorylation of β-catenin is inhibited, leading to the accumulation of β-catenin in the cytoplasm and nucleus. The nuclear accumulation of β-catenin mediates the transcriptional activation of the TCF/LEF and WNT responsive genes [16,47]. When there is limited or no WNT ligand, β-catenin is degraded by the proteasome after being phosphorylated by a GSK3β-AXIN-CK1-APC destruction complex. Ultimately, the levels of β-catenin at the intercellular junctions and in the nucleus determine the activation of WNT responsive genes and the biological response of the cells. Complicating this further is that the level of WNT/β-catenin signalling is also precisely modulated by the binding of WNT ligands to inhibitors such as DKK1, DKK2, SOST, SOSTDC1 KREMEN1, and KREMEN2 [16], and the binding of WNT inhibitors to LRP5 and LRP6 co-receptors [16,45,46].

Here, we report the first description of heterozygous *APC* variants in patients with mesiodentes or an isolated supernumerary tooth. Two unrelated Thai patients (patients 1 and 2) carried the same rare missense variant (p.Val1125Ala), while one patient (patient 6) with mesiodens carried a compound heterozygous mutation (p.Cys914Gly and p.Asn1908Tyr) in *APC*. APC is a tumour-suppressor protein and its loss-of-function mutations have been demonstrated to result in the over-activation of WNT/β-catenin signalling, and familial adenomatous polyposis syndrome with or without supernumerary tooth formation [35,39].

APC loss-of function or constitutive β-catenin activation in adult dental tissue has previously been shown to result in new tooth formation [39,48]. The process is non-cell autonomous. Only a small number of APC-deficient cells was reportedly sufficient to induce the surrounding wild-type epithelial and mesenchymal cells to collaborate in forming the new teeth [39]. Notably, supernumerary incisors were formed in adult mouse oral tissues in response to epithelial APC loss-of-function or Wnt/β-catenin over-activation [39].

### 3.1. APC Protein Structure and Possible Impact of Identified Variants on APC Function

APC is a protein with multiple functionally characterized domains (Figure 4). It consists of multiple regions important for oligomerization, an armadillo repeat region, a series of 15- and 20-amino acid repeats, an AXIN-binding domain, and a C-terminus that contains a basic domain and binding sites for End-Binding protein 1 (EB1) and the human Discs Large (HDLG) protein [42,49]. Each of these domains can serve as binding sites for one or more different partner proteins, including those of the Wnt signaling complex (β-catenin, AXIN, and KIF3a), the cytoskeletal regulators including of EB1, HDLG, and IQGAP1, the Rac guanine-nucleotide-exchange factor (GEF), Asef1, and CTBP [26,42,50]. 

Each of the identified variants can feasibly impact APC function in various ways but with a similar impact on Wnt signaling. The p.Val1125Ala change is located in the 15-amino acid repeat region that is required for binding to CTBP. Reduced CTBP interaction strength would be predicted to result in increased β-catenin, leading to the over-activation of WNT/β-catenin signalling, thus explaining subsequent supernumerary tooth formation. The association of this rare variant in two unrelated patients with supernumerary teeth supports its pathogenicity. 

Some of the other variants are also predicted to lead to increased Wnt/β-catenin signalling but through reduced interaction with other protein partners. Based on the location of the p.Ile2043Val change—in a highly conserved residue in the APC SAMP3 motif (Figure 5) that is directly involved in contact with the RGS domain of AXIN—we would predict a reduced interaction with AXIN, rather than with CTBP. The result of such a disruption would likely impact the proper nucleation of actin, with a potential indirect impact on the signalling. In contrast, the p.Ala2795Thr variant may perturb the interaction with EB1 and/or self-association (Figure 5). EB1 is a protein that binds to the plus end of microtubules and plays a critical role in microtubule dynamics [51]. Reduced the binding of APC to EB1 would be predicted to lead to increased nuclear β-catenin, and, thus, the elevation of WNT/β-catenin signalling. 

In patient 6, either or both rare missense variants could contribute to the presentation of mesiodens. The p.Cys914Gly variant could have an effect on the APC multimerization and, thus, the assembly of the larger destruction complex. In contrast, the p.Asn1908Tyr variant (Figure 4)—a completely novel variant—resides in a highly charged inter-repeat region and changes from the neutral polar asparagine (Asn) to a hydrophobic non-polar residue, tyrosine (Tyr). Such a significant change might be sufficient to disrupt its binding with either AXIN or b-catenin, leading in either case to impaired β-catenin degradation and the over-activation of Wnt/β-catenin signalling.

### 3.2. APC Mutations, Ciliary Dysfunction, and Supernumerary Tooth Formation

Primary cilia are crucial for the integration of Hh and Wnt/β-catenin signalling and both pathways are important for tooth development [52,53,54]. The dysregulation of SHH and Wnt/β-catenin signalling pathways are implicated in the malformation of teeth [55]. The EB1 and kinesin-family-member 3A (KIF3A) are APC-binding proteins that are involved in intraflagellar transport [26]. KIF3A, an anterograde intraflagellar transport motor protein, is important for tooth development because it regulates the integrity of primary cilia and various cellular functions including the differentiation of stem cells involved in tooth development via the WNT pathway [56]. The loss of *Kif3a* in mice results in the over-activation of WNT signalling and subsequent dental anomalies [52]. Therefore, supernumerary tooth formation as a result of *APC* mutations implicates ciliary dysfunction as an underlying pathogenetic mechanism [26,52]. 

Lastly, we are convinced that these *APC* variants contributed to the supernumerary tooth phenotypes in the patients because no other rare variants in the known dental anomalies-associated genes (including *WNT10A, WNT10B, LRP4, LRP5, LRP6, DKK1, PAX9, AXIN2, MSX1, WLS, BMP4, GREM2, TFAP2B, TSPEAR, PITX2, EVC, EVC2, COL1A2, ANTXR1, FGF10, SMOC2, KREMEN1, KDF1, ATF1, DUSP10, EDA, EDAR, EDARADD,* and CASC8 [19,20,21,22,23] were identified in any of these patients.

## 4. Materials and Methods

### 4.1. Patient Recruitment

**Figure 6:**
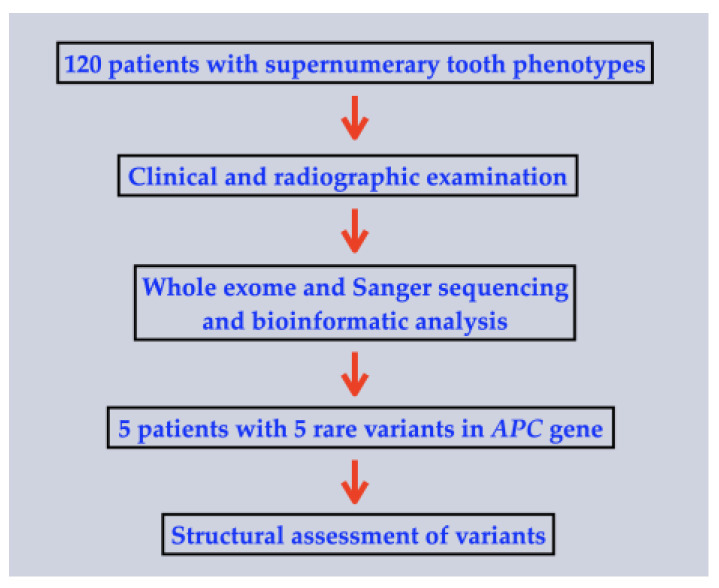
Flowchart describing the methodology of the study.

This study was conducted in accordance with the Declaration of Helsinki and national guidelines. Informed consent was obtained from the patients or the parents of the patients in accordance with the regulations of the Human Experimentation Committee of the Faculty of Dentistry, Chiang Mai University (certificate of approval number 71/2020). The inclusion criteria were patients with isolated supernumerary tooth phenotypes including, mesiodens. The exclusion criteria were patients with a normal number of teeth. Oral and radiographic examinations (panoramic or periapical radiographies) were performed on the cohort of 120 patients affected with various kinds of isolated extra tooth phenotypes, which included 93 patients with isolated mesiodentes and 27 (17 males; 10 females) patients with isolated supernumerary teeth (Figure 6). 

Regarding 93 patients with mesiodentes, 63 (68%) were males and 30 (32%) were females. Seventy-seven patients (82.8%) had single mesiodentes, while 16 (17.2%) of them had double mesiodentes. The orientation of the mesiodentes was noted in 63 mesiodentes of 53 patients; 42 (66.7%) of them had normal orientation, 20 (31.7%) were inverted, and 1 (1.6%) had transverse orientation. The eruption status had been noted in 59 mesiodentes of 48 patients: 31 (52.5%) erupted and 28 (47.5%) were unerupted. For the patients with isolated supernumerary teeth that were not mesiodens or mesiodentes, the information of the supernumerary teeth was not available. 

### 4.2. Whole Exome Sequencing, Mutation Analysis, and Bioinformatic Analyses

All the consented patients’ genomic DNA was isolated from saliva using Oragene-DNA (OG-500) Kit (DNA Genotek, Ottawa, Ontario, Canada). Whole exome sequencing (WES) was performed for 120 patients with isolated mesiodentes or isolated supernumerary teeth (Macrogen Inc, Seoul, Korea). It was also performed on some of the unaffected siblings and unaffected parents (if available). The average depth of the sequencing was 80× using the targeted capture SureSelect V6 kit (PR7000-0152; Agilent Technologies, Santa Clara, CA, USA), which also captures untranslated regions. We adopted GATK3.8 best practices to identify variants for each sample; the alignment of the raw sequencing FASTQ file with the human genome reference sequence, GRCh38+decoy, was performed using BWA-mem. The variant effect predictor (VEP) and the database of nonsynonymous functional prediction (dbNSFP) were used to computationally assign effects to the resulting variants of each individual. The annotated variant calling format (VCF) files were stored in our in-house database that allows us to query pathogenic variants according to different modes of segregation. Furthermore, the variant allele frequencies were determined by comparing against public databases, including 1000 Genomes, gnomAD, GenomeAsia, and the recent Thai Reference Exome database. The prioritization of the variants was established based on multiple considerations: (1) whether the gene harbouring each variant has an established role in tooth development and/or was previously implicated in dental anomalies; (2) the allele rarity; (3) CADD score > 15; and (4) localization to or near an important functional region of the protein. The selected variants of interest were then validated and, if familial samples available, tested for segregation using PCR-based amplification and Sanger sequencing. The Sanger sequencing was performed to confirm the variants. The sequence primers used were as follows: Exon 16a, forward: 5′-TGGGCAAGACCCAAACACAT-3′; reverse: 5′-TGGATGGAGCTGATTCTGCC-3′. Exon 16b, forward: 5′-AGGGGCAGCAACTGATGAAA-3′; reverse: 5′-GCAGCAGCAGCTTGATGTAA-3′. Exon 16c, forward: 5′-GGGTAATGGCAGTGTTCCCA-3′; reverse: 5′-GTAAGACCCAGAATGGCGCT-3′. Exon 16d, forward: 5′-AGTTTGGAGAGAGAACGCGG-3′; reverse: 5′-GTCGGCTGGGTATTGACCAT-3′. Exon 16e, forward: 5′-TTCACCTCATCATTACACGCCT-3′; reverse: 5′-TCAGGGGGCTCAGTCTCTTT-3′.

### 4.3. Structural Assessment of Variants

An available crystal structure of the human APC SAMP3 domain in complex with AXIN (1emu.pdb; [41]) was retrieved from the Protein Data Bank (www.rcsb.org) (accessed on 8 January 2023) and investigated using PyMol visualization software (Schrödinger Inc, New York, NY, USA).

## 5. Study Limitation

Our cohort consisted of 120 patients with supernumerary tooth phenotypes. However, we only had DNA samples and dental information of the affected patients who came for oral and radiographic examinations. We are aware that it would have been ideal if we had the unaffected family members of each patient participate in the study in order to study the co-segregation between the phenotype and genotype. It would also have strengthened the relationship of the heterozygous variants in the *APC* gene and the supernumerary tooth phenotypes. In some patients, mesiodens was an incidental finding。 Most patients came for dental check-up or treatments of dental caries. Therefore, the use of cone beam computed tomography (CBCT) was not indicated. The use of CBCT would have shown the better morphology of mesiodentes. However, the use of CBCT would have been over-treatment for the patients who came to our dental clinic for a check-up. Notably, approximately 50% of mesiodentes are unerupted; therefore, the people who carried *APC* variants in the studied population of gnomAD, might not be dentally “normal” as they might have unerupted mesiodentes. 

## 6. Conclusions

The activation of Wnt/β-catenin signalling is a prerequisite for odontogenesis [39]. In the normal situation, APC, a member of the AXIN-CK1-GSK3β-APC destruction complex, functions to modulate Wnt/β-catenin signalling to establish regular teeth number and positions. The presence of supernumerary teeth in the patients with APC-associated familial polyposis syndrome indicates the direct relationship of *APC* variants and supernumerary tooth formation. The association of rare *APC* variants with isolated supernumerary tooth formation in multiple individuals supports a contributory, if not, causal role in the formation of mesiodentes and a supernumerary tooth. 

## Figures and Tables

**Table 1:** Patients with *APC* variants and their dental phenotypes.

Families	Patients	Phenotypes	*APC* Variants NM_000038.6; NP_000029.2	Prediction/Ranking
1	1 (Female)	Supernumerary erupted mandibular left premolar	c.3374T>C p.Val1125Ala chr5-112174665-T-C rs377278397 MAF: 0.0005993	MutationTaster: Disease causing Prob = 0.944130296883564 Polyphen-2: Benign; score = 0.001 SIFT: Tolerated; score = 0.244 CADD-PHRED score = 21.7 DANN: Benign; score = 0.9507
2	2 (Male)	Mesiodens (Double with normal orientation)		
3	3 (Female)	Mesiodens (Single; erupted)	c.6127A>G p.Ile2043Val chr5-112177418-A-G rs876660233 MAF: 0.000007085	MutationTaster: Disease causing Prob = 0.999923872840111 Polyphen-2: Probably damaging; score = 0.958 SIFT: Damaging; score = 0.011 CADD-PHRED score = 24.9 DANN score = 0.9983
4		Normal mother	No variants	MutationTaster: Disease causing Prob = 0.999223891277048 Polyphen-2: Probably damaging; score = 0.936 SIFT: Tolerated; score = 0.068 CADD-PHRED score = 22.2 DANN score = 0.9909
	4 (Male)	Mesiodens (Double; unerupted & inverted)	c.8383G>A p.Ala2795Thr chr5-112179674-G-A rs369264968 MAF:0.00004400	
	5 (Male)	Mesiodens (Double; both were erupted)		
		Normal sister	No variants	
5	6 (Male)	Mesiodens (Double; unerupted) One is inverted, the other had normal orientation	Variant 1 c.2740T>G p.Cys914Gly chr5-112174031-T-G rs1554084426 Not reported in gnomAD Variant 2 c.5722A>T p.Asn1908Tyr chr5-112177013-A-T No rs number Not reported in gnomAD	Variant 1 MutationTaster: Disease causing Prob = 0.99929839701033 Polyphen-2: Benign score = 0.055 SIFT: Tolerated; score = 0.127 CADD-PHRED score = 21.5 DANN score = 0.8956 Variant 2 MutationTaster: Polymorphism Prob = 0.999999988244843 Polyphen-2: Benign score = 0.214 SIFT: Damaging; score = 0.046 CADD-PHRED score = 17.19 DANN score = 0.9609

## Data Availability

Not applicable.
